# More Than Skin Deep: An Unusual Case of a Migrating Pacemaker Lead

**DOI:** 10.7759/cureus.34405

**Published:** 2023-01-30

**Authors:** Rami H Mahmoud, Brooke A Hensley

**Affiliations:** 1 Medicine, University of Miami Miller School of Medicine, Miami, USA; 2 Emergency Medicine, Jackson Memorial Hospital, Miami, USA

**Keywords:** pacemaker lead migration, pacemaker lead, pacemaker failure, pacemaker, pacemaker lead perforation

## Abstract

Pacemakers are commonly utilized in clinical practice and are generally well tolerated; therefore, clinicians may not be exposed to potential complications associated with pacemakers. This case report aims to illustrate the clinical presentation of a pacemaker lead migration, an uncommon potential complication. We present an 83-year-old male with a past medical history of complete atrioventricular block managed with a permanent pacemaker who presented with an open wound on his right chest. He had capped, abandoned right-sided leads from a previous pacemaker. At presentation, there was blood-tinged, yellow drainage and visible erosion of his electrodes. Computed tomography showed the right ventricular pacing lead perforating the right ventricle. Pacemaker lead migration outside of the chest wall is rare. Perforations may present asymptomatically or strikingly with effusions, pneumothoraces, hemothoraces, or cardiac tamponade. Management options include lead repositioning or extraction.

## Introduction

Pacemakers are commonly utilized in clinical practice. In the United States, around 600,000 pacemakers are placed annually [1]. They are generally well tolerated, so many clinicians may not be exposed to potential complications associated with pacemakers. This article aims to illustrate an uncommon potential pacemaker lead complication. We hope that this exposure will lead to more timely detection and treatment for patients presenting with pacemaker lead perforations. Furthermore, we hope to educate society at large about this complication to promote greater detection and better outcomes for our patients experiencing pacemaker lead perforations.

## Case presentation

**Figure 1:**
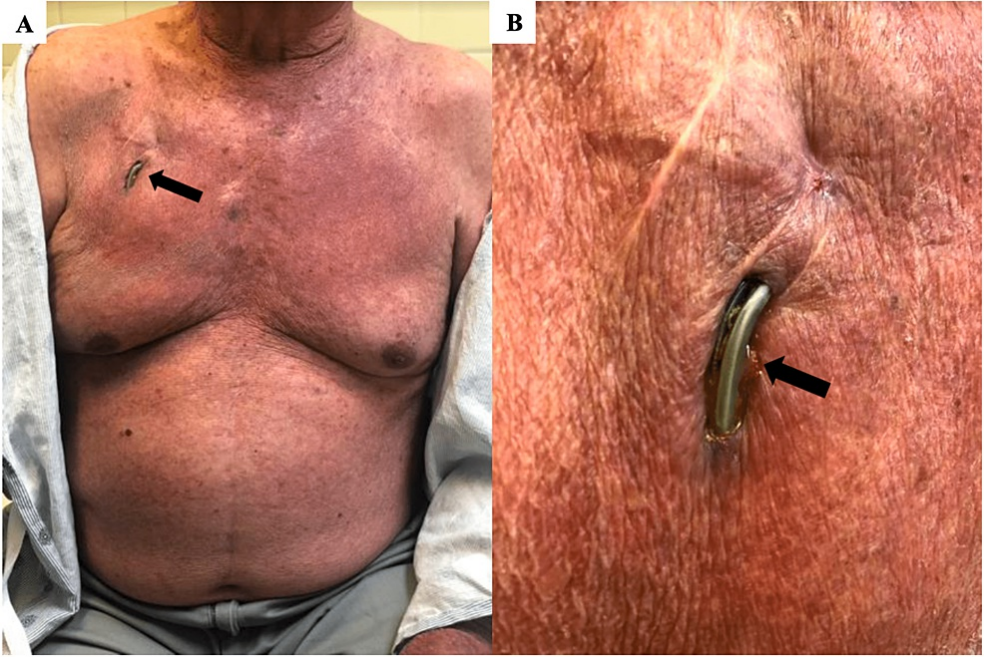
Regular (A) and zoomed-in (B) view of the migrated right ventricular pacing lead outside of the chest wall (arrows)

**Figure 2:**
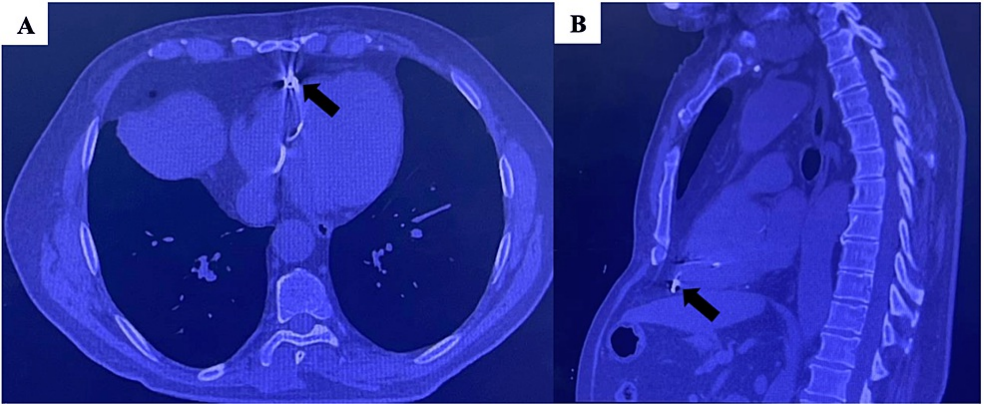
Axial (A) and sagittal (B) views of chest computed tomography showing the right ventricular pacing lead perforating the right ventricle (arrows)

An 83-year-old man with a past medical history of hypertension, type 2 diabetes, chronic kidney disease, and complete atrioventricular block managed with a permanent pacemaker presented to the cardiologist with an open wound on his right chest that he noticed over the past six months. He underwent dual-chamber pacemaker implantation on the right side 11 years prior. After several years, the leads became detached from the generator. As a result, the generator was removed three years earlier, and the right-sided leads were abandoned and capped. A new permanent pacemaker was subsequently placed on the left side. The patient presented to the emergency department (ED) with new-onset blood-tinged, yellow drainage and obvious erosion of his electrodes (Figure *1*). Computed tomography without contrast showed the right ventricular pacing lead perforating the right ventricle (Figure 2).

Our patient underwent lead removal by laser transvenous extraction; however, the pacemaker was left in place. He developed hypotension intraoperatively, and a transesophageal echocardiogram demonstrated a pericardial effusion. A pericardial window was performed showing only clear fluid. A pathology report of surrounding bone and cartilage showed no specific pathologic changes, and blood cultures were negative. Intraoperative site cultures were not obtained, but the decision was made to start antibiotics for a suspected pocket infection. The patient was recommended to receive vancomycin via a peripherally inserted central catheter (PICC) line but refused this recommendation. He was subsequently started on linezolid at the time of discharge and had an uncomplicated recovery.

## Discussion

There are three basic types of pacemakers, each of which involves different lead positionings. One lead is placed in either the right atrium or the right ventricle in single-chamber pacemakers, whereas one lead is placed in the right atrium and one in the right ventricle in dual-chamber pacemakers. Biventricular pacemakers involve lead placement in the right atrium, right ventricle, and left ventricle. For all types, the pulse generator is generally placed subcutaneously beneath the clavicle on the patient’s nondominant side. Pacemaker implantation remains at low risk in its potential complications; however, post-implant effusions, cardiac tamponade, and even death are possible [2]. Specifically, myocardial perforation due to a pacemaker lead is an uncommon phenomenon, with a frequently cited incidence of less than 1% [3-5]. Perforations may occur acutely at the time of implantation or, very rarely, more than one month after implantation as late lead perforations (LLP) [6]. Migration of a lead outside of the chest wall following an LLP, as in this case, is especially rare. Patients with LLP may present with a range of symptoms including shortness of breath due to pericardial effusions and cardiac tamponade, and in cases of migration into the pulmonary system, shortness of breath may result from conditions like pneumothorax and hemothorax [6]. Swelling and discomfort over the pacemaker pocket, sensations of shocks, and hiccups are also reported [6]. In some instances, patients may present asymptomatically or subacutely with nonspecific symptoms such as fatigue and dizziness [4,6].

Definitive management options include simple repositioning of the lead or extraction, which may be done as an open chest lead extraction or percutaneous lead extraction [6]. Post-implantation, lead repositioning may become increasingly complicated as time elapses due to the formation of fibrotic adhesions [6]. In cases of lead extractions, life-threatening complications such as cardiac tamponade may occur [6].

## Conclusions

This is an interesting case demonstrating that the gravity of the problem lies beyond the superficial presentation. The patient was otherwise asymptomatic and presented to the ED for evaluation years after the pacemaker was inserted. Many patients have financial constraints that limit their access to healthcare via the ED, so it is imperative to recognize that seemingly superficial complications could be the tip of the iceberg. Chest wall migration of a pacemaker lead must prompt a provider to pursue emergent CT imaging to investigate the extent of pacemaker malfunction, even in an asymptomatic patient, as in this case.
